# Overlapping and Specific Functions of the Hsp104 N Domain Define Its Role in Protein Disaggregation

**DOI:** 10.1038/s41598-017-11474-9

**Published:** 2017-09-11

**Authors:** Jungsoon Lee, Nuri Sung, Jonathan M. Mercado, Corey F. Hryc, Changsoo Chang, Sukyeong Lee, Francis T. F. Tsai

**Affiliations:** 1 Verna and Marrs McLean Department of Biochemistry and Molecular Biology, Baylor College of Medicine, One Baylor Plaza, Houston, TX 77030 USA; 2 Department of Molecular and Cellular Biology, Baylor College of Medicine, One Baylor Plaza, Houston, TX 77030 USA; 3 Graduate Program in Structural and Computational Biology and Molecular Biophysics, Baylor College of Medicine, One Baylor Plaza, Houston, TX 77030 USA; 4 Department of Molecular Virology and Microbiology, Baylor College of Medicine, One Baylor Plaza, Houston, TX 77030 USA; 5 Center for Drug Discovery, Baylor College of Medicine, One Baylor Plaza, Houston, Texas 77030 USA; 6 Structural Biology Center, Biosciences Division, Argonne National Laboratory, 9700 South Cass Avenue, Argonne, IL 60439 USA

## Abstract

Hsp104 is a ring-forming protein disaggregase that rescues stress-damaged proteins from an aggregated state. To facilitate protein disaggregation, Hsp104 cooperates with Hsp70 and Hsp40 chaperones (Hsp70/40) to form a bi-chaperone system. How Hsp104 recognizes its substrates, particularly the importance of the N domain, remains poorly understood and multiple, seemingly conflicting mechanisms have been proposed. Although the N domain is dispensable for protein disaggregation, it is sensitive to point mutations that abolish the function of the bacterial Hsp104 homolog *in vitro*, and is essential for curing yeast prions by Hsp104 overexpression *in vivo*. Here, we present the crystal structure of an N-terminal fragment of *Saccharomyces cerevisiae* Hsp104 with the N domain of one molecule bound to the C-terminal helix of the neighboring D1 domain. Consistent with mimicking substrate interaction, mutating the putative substrate-binding site in a constitutively active Hsp104 variant impairs the recovery of functional protein from aggregates. We find that the observed substrate-binding defect can be rescued by Hsp70/40 chaperones, providing a molecular explanation as to why the N domain is dispensable for protein disaggregation when Hsp70/40 is present, yet essential for the dissolution of Hsp104-specific substrates, such as yeast prions, which likely depends on a direct N domain interaction.

## Introduction

The capacity of cells to tolerate a variety of stress conditions is essential for organismal health. To protect cells from damage, molecular chaperones assist in the folding of other proteins, thereby providing the first line of defense against protein misfolding and aggregation^1^. However, the majority of stress-inducible molecular chaperones does not recognize or remodel protein aggregates. Instead, cells have evolved powerful ATP-driven protein disaggregases that have the remarkable ability to rescue stress-damaged proteins from an aggregated state^2–4^.

**Figure 1:**
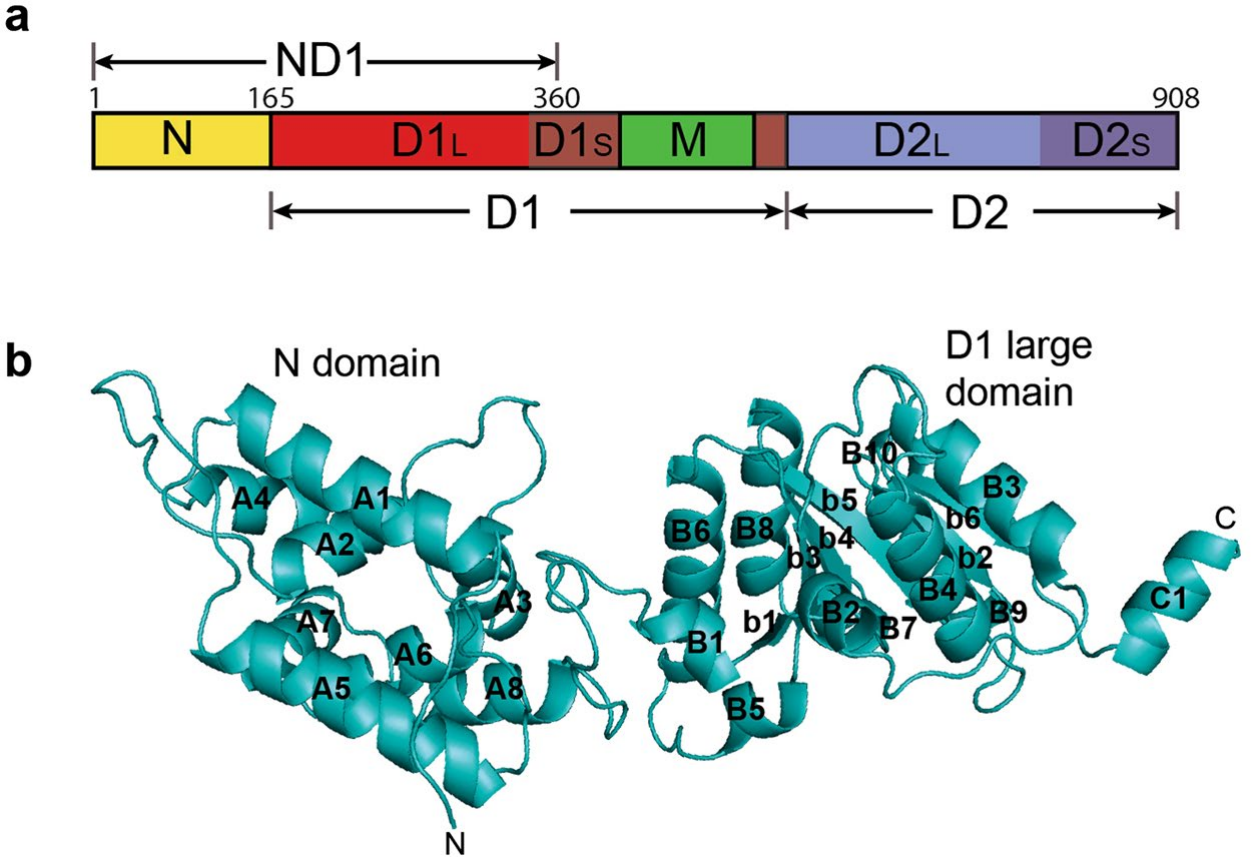
Crystal structure of Hsp104_ND1_. (**a**) Schematic diagram of the Hsp104 domain organization. (**b**) Ribbon diagram of Hsp104_ND1_. Secondary structural elements are labeled according to their domain assignment: N domain (A), D1-large domain (B), and D1-small domain (C). An upper case letter designates an α-helix and a lower case letter a β-strand.

Members of the ring-forming Hsp104 family are the principal protein disaggregases in fungi (Hsp104), plants (Hsp101), and eubacteria (ClpB), but are absent in animal cells^5^. Hsp104 chaperones are multi-domain proteins consisting of an N domain, an M domain, and two AAA+ type nucleotide-binding domains, termed D1 and D2, which can be subdivided into a larger α/β domain (D1- and D2-large) and a smaller α-helical domain (D1- and D2-small) (Fig. 1a). The M domain serves an important regulatory function^6^ and mediates the physical interaction with Hsp70^7,8^. The 3D structures of the isolated Hsp104 N domain^9^ and of full-length Hsp104^10^ have been determined, including that of their hexamer assembly in different nucleotide states^11–13^.

It is now widely accepted that Hsp104 recognizes amorphous and ordered protein aggregates^14–16^, and extracts polypeptides that are then threaded through the Hsp104 hexamer by pore loops in the D1 and D2 domains^17,18^, thereby coupling the energy of ATP binding and hydrolysis to drive protein unfolding. Although Hsp104 is a functional ATPase, efficient protein disaggregation depends on the functional cooperation and physical interaction with the Hsp70 system (Hsp70/40), which directs the Hsp104 hexamer to amorphous aggregates and yeast prions *in vivo* ^14,15,19^, activates the Hsp104 motor in a species-specific manner^7,8,20,21^, and assists in the recovery of Hsp104 substrates that depend on Hsp70/40 for folding^11,21,22^. Although the Hsp104:Hsp70/40 bi-chaperone system represents the functionally active form *in vivo*, the Hsp104 protein-remodeling activity can also be elicited *in vitro* in the absence of Hsp70/40 by mutation or under chemically defined nucleotide conditions that asymmetrically decelerate the ATPase activity of Hsp104^23^. Additionally, modification of the M domain by point mutation^24,25^ or by insertion of T4 lysozyme within the M domain helix 2 (Hsp104_T4L_)^7,11^ renders Hsp104 constitutively active for protein disaggregation and independent of Hsp70/40 chaperones in a substrate-dependent manner.

Despite major advances in our mechanistic understanding of Hsp104 function, it remains poorly understood how Hsp104 recognizes and binds protein substrates of diverse sequence, shape, and size. In this context, the importance of the N domain to Hsp104 function is perhaps most controversial. Initially proposed to be essential for ClpB function^26,27^ by mediating substrate interaction^26,28–31^, it was later shown that the N domain is dispensable for protein disaggregation *in vitro* ^32,33^ and thermotolerance development *in vivo* ^33,34^. Although the N domain appears to be dispensable for protein disaggregation, it was shown more recently that the N domain orientation can affect protein disaggregation^35^ and abolishes ClpB-dependent protein disaggregation when mutated^36^. Consistent with an important but non-essential role in eubacteria, the N domain of *Saccharomyces cerevisiae* Hsp104 is dispensable for thermotolerance development and yeast prion replication^14,37^, as well as for protein binding and recovery *in vitro* and *in vivo* ^38^. However, the Hsp104 N domain enhances protein disaggregation *in vitro* ^7^, mediates the interaction with the prion form of Sup35p^15^, and is essential for yeast prion dissolution^16^ and curing by Hsp104 overexpression^37^.

Delineating the functional role of the non-essential N domain is difficult because of the close physical and synergistic interaction between Hsp104 and the cognate Hsp70 chaperone system. Like Hsp104, Hsp70 is a functional ATPase that chaperones misfolded and aggregation-prone proteins. Because Hsp70 binding to the M domain is essential to unleash the protein disaggregating activity of Hsp104, Hsp104 mutants that abolish protein disaggregation could be impaired in substrate interaction, in Hsp70 cooperation, or both. Here, we present the crystal structure of an *S*. *cerevisiae* Hsp104 N-terminal fragment (residues 1–360) consisting of the N domain and an extended D1-large domain. We find that the N domain is bound to a C-terminal helical segment of the neighboring D1 domain, mimicking substrate interaction. We show by structure-guided mutagenesis that the pseudo two-fold symmetrical N domain features a bipartite peptide-binding interface, involving the first helix of each N domain repeat (helix A1 and A5). Hsp104 variants featuring point mutations in either interface are defective in protein disaggregation. While helix A5 mutants are impaired in Hsp70 interaction, mutations in helix A1 are defective in substrate binding and can be rescued by Hsp70/40 chaperones. We propose that the N domain plays an important role in protein disaggregation by facilitating both substrate binding and Hsp70 interaction to accommodate a variety of Hsp104 substrates.

## Results

### Crystal Structures of Hsp104_ND1_

To provide a molecular understanding of the functional role of the Hsp104 N domain, we solved the 2.8 Å resolution crystal structure of an N-terminal fragment of yeast Hsp104 (residues 1–360; Hsp104_ND1_) comprising the N domain and an extended D1-large domain featuring the first α helix of the D1-small domain, termed the C1 helix (residues 343–351)^39^ (Fig. 1a). Despite a sequence identity of 42% between yeast Hsp104 and bacterial ClpB (see Supplementary Fig. S1), no structure solution could be found by molecular replacement using the previously determined crystal structure of *Thermus thermophilus* ClpB (PDB ID: 1QVR)^39^ as a search model, even when performing the analysis with the individual N and D1 domains. To solve the phase problem, we crystallized an engineered Hsp104_ND1_ construct featuring three methionine substitutions at Leu36, Phe118, and Leu248 (Hsp104_MMM_), and determined the crystal structure of this variant by seleno-methionine (SeMet) MAD phasing (Table 1). The unbiased experimental map enables tracing of residues 4 to 352 (Fig. 1b). No unaccounted electron density was observed that could be attributed to bound nucleotide, irrespective of the 5 mM nucleotide (ADP or ADPNP) that was added during crystallization, suggesting that Hsp104_ND1_ was crystallized in the nucleotide-free state and indicating that a complete AAA+ domain featuring both the D1-large and -small domains are needed for nucleotide binding.

**Table 1:** Data collection, MAD phasing, and refinement statistics.

	Native	Se-Met (Hsp104_MMM_)		
**Data Collection Statistics**				
Space group	*P* 6_5_22	*P* 6_5_22		
Unit Cell	*a* = 179.1 Å, *b* = 179.1 Å, *c* = 69.7 Å	*a* = 179.5 Å, *b* = 179.5 Å, *c* = 69.1 Å		
	α = 90°, β = 90°, γ = 120°	α = 90°, β = 90°, γ = 120°		
**Source**	**NSLS-X25**	**NSLS-X25**		
Wavelength (Å)	λ = 1.10	λ_1_ = 0.9789	λ_2_ = 0.9792	λ_3_ = 1.0024
Resolution (Å)	41.53–2.82	44.8–3.5	44.8–3.5	44.8–3.8
Completeness (%)^a^	84.2 (12.7)	99.8 (100)	99.8 (99.5)	99.9 (99.9)
Redundancy	14.7 (1.7)	6.9 (6.8)	6.9 (6.5)	6.7 (6.9)
R_sym_^a,b^	0.076 (0.386)	0.086 (0.393)	0.091 (0.497)	0.088 (0.320)
I/σ^c^	17.6	12.9	11.1	10.7
**MAD Phasing Statistics**				
R_iso_^d^	0.222		0.044	0.053
R_Cullis_^e^			0.62	0.72
Phasing Power^f^		2.38	2.07	1.16
Figure of Merit^g^		0.63 (centric)	0.60 (acentric)	
**Refinement Statistics**				
Resolution (Å)	41.53–2.82			
No. reflections	14036			
R_cryst_/R_free_	0.210/0.279			
No. atoms	2725			
Protein	2721			
Water	4			
B-factors	94.1			
Protein	94.1			
Water	74.3			
rmsd bond (Å)	0.002			
rmsd angle (°)	0.399			
**Ramachandran**				
Favored (%)	97.1			
Outliers (%)	0.0			

^a^Values for the highest resolution shell are given in parentheses. ^b^R_sym_ = Σ_hkl_|I(hkl) − < I(hkl) > |/Σ_hkl_I(hkl), where <I(_hkl_)> is the mean of the symmetry equivalent reflections of I(hkl). ^c^Based on unmerged data. ^d^R_iso_ = Σ|F_PH_ − F_P_|/ΣF_p_, where F_p_ is the peak (λ_1_), and F_PH_ are the inflection (λ_2_), low energy remote (λ_3_) or native structure factor amplitudes. ^e^R_Cullis_ = Σ||F_PH_ ± F_P_| − F_H_^calc^|/Σ|F_PH_ ± F_P_| for centric reflections only. ^f^Isomorphous phasing power = Σ|F_H_|/Σ||F_PH_^obs^| − |F_PH_^calc^||; anomalous phasing power = Σ|F″_H_|/Σ||AD_obs_| − |AD_calc_||. ^g^Figure of Merit = weighted mean of the cosine of the deviation from α_best_.

The 3D structure of our N and D1-large domains is nearly identical to those of the isolated *S*. *cerevisiae* Hsp104 N domain^9^ and *Chaetomium thermophilum* Hsp104 D1-large domain^10^ that were recently reported, and superimpose pair-wise with an RMSD of only 0.60 ± 0.09Å^2^ (N domain) and 1.59 ± 0.12 Å^2^ (D1-large domain). The N domain can be divided into two repeats related by a pseudo two-fold symmetry. However, unlike bacterial ClpA/B proteins, the N domain of Hsp104 features a four amino acid insertion that extends the length of an intervening loop region (residues 40–49) conferring asymmetry to the yeast N domain structure (Fig. 1b). The structure of the extended D1-large domain shares the canonical α/β-fold of bacterial Hsp100 AAA+ ATPases (Fig. 1b), featuring the conserved Walker A, Walker B, sensor-1, and arginine (Arg)-finger motifs required for ATP binding and hydrolysis^40^ (see Supplementary Fig. S1).

### The crystal structure of the N domain features a peptide-binding site

**Figure 2:**
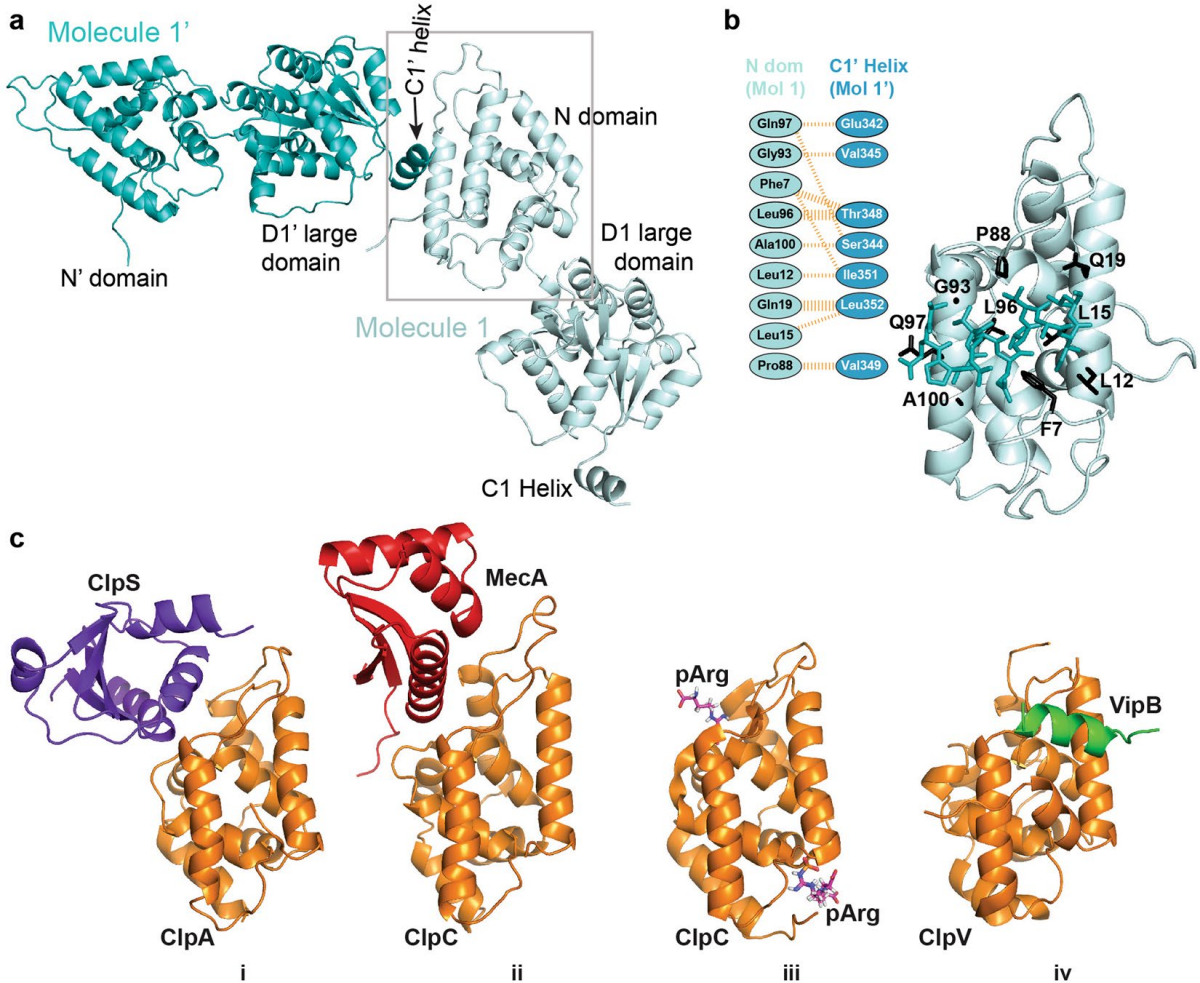
The substrate-binding interface of the Hsp104 N domain. (**a**) Molecular content of two asymmetric units showing the interaction between the N domain of one molecule and the neighboring C1 helix (C1′). (**b**) Specific interactions between the N domain and the C1′ helix. N domain residues in contact with the C1′ helix are shown as stick model. The substrate-binding interface consists of hydrophobic interactions. Molecular interactions between the N domain and the neighboring C1 helix were analyzed using PDBsum^54^. The thickness of the orange dashed line correlates with the number of interactions made. (**c**) The substrate-binding site of the Hsp104 N domain is distinct from protein-binding interfaces in other Hsp100 proteins. The orientation of Hsp100 N domains is similar to that shown in Fig. 2b. (i) N domain of ClpA bound to its adaptor ClpS^42^, (ii) ClpC bound to its adaptor MecA^43^, and (iii) ClpC bound to a phosphorylated arginine mimicking substrate interaction^44^, and (iv) ClpV bound to a segment of its VipB substrate^45^.

Surprisingly, we find that the N domain of Hsp104_ND1_ interacts with the C1 helix of the neighboring D1-small domain (Fig. 2a,b and Supplementary Fig. S2). Binding interactions are hydrophobic in nature consistent with the notion that molecular chaperones bind misfolded and aggregation-prone protein conformers that expose hydrophobic residues on their surface^41^. N domain segments involved in peptide binding include helix A1 (Leu12, Leu15, and Gln19), helix A5 (Gly93, Leu96, Gln97, and Ala100), and additional hydrophobic residues (Phe7 and Pro88) (Fig. 2b). Notably, this peptide-binding site differs from binding sites reported for ClpA^42^, ClpC^43,44^, and ClpV^45^ (Fig. 2c), but overlaps with the proposed peptide-binding site recently reported for ClpB^36^. However, to our knowledge, this is the first time that an interaction with a helical peptide is observed.

**Figure 3:**
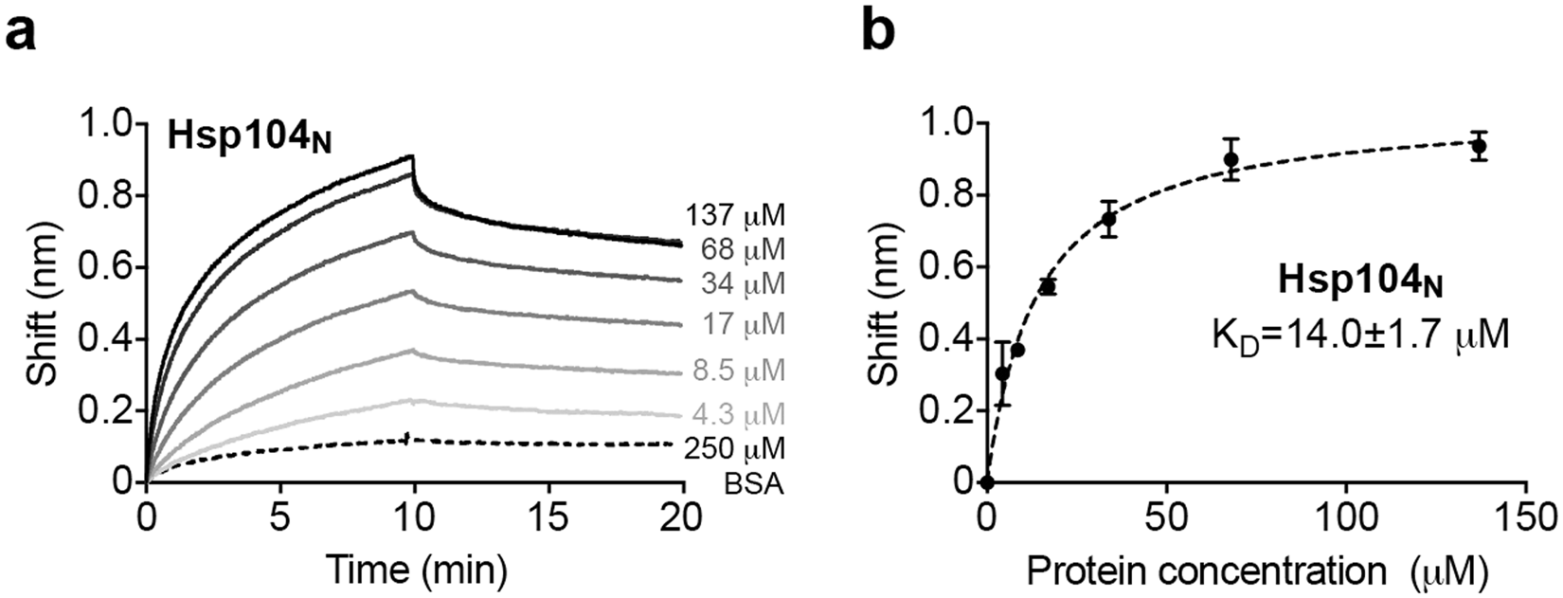
Direct binding of Hsp104_N_ to the C1 peptide determined by biolayer interferometry. (**a**) Binding of Hsp104_N_ (solid lines) or BSA (dotted line) to immobilized biotinylated C1 peptide was measured by light distance shift (nm). A representative curve set of association (0–10 min) and dissociation (10–20 min) curves for various Hsp104_N_ concentrations (4.3 to 137 µM) or BSA control (250 µM) from two independent measurements are shown. (**b**) Averaged equilibrium light distance shift at different Hsp104_N_ protein concentrations from two independent measurements.

To confirm that this Hsp104 N domain-peptide interaction is also observed in solution, we synthesized a peptide with its sequence derived from the C1 helix (GEVAEPSVRQTVAILRGLQ) and measured the binding interaction using biolayer interferometry (Fig. 3a,b). Our results show that the C1 peptide bound to the isolated N domain with a K_D_ of 14.0 ± 1.7 μM.

### The N domain contributes towards protein disaggregation when present

**Figure 4:**
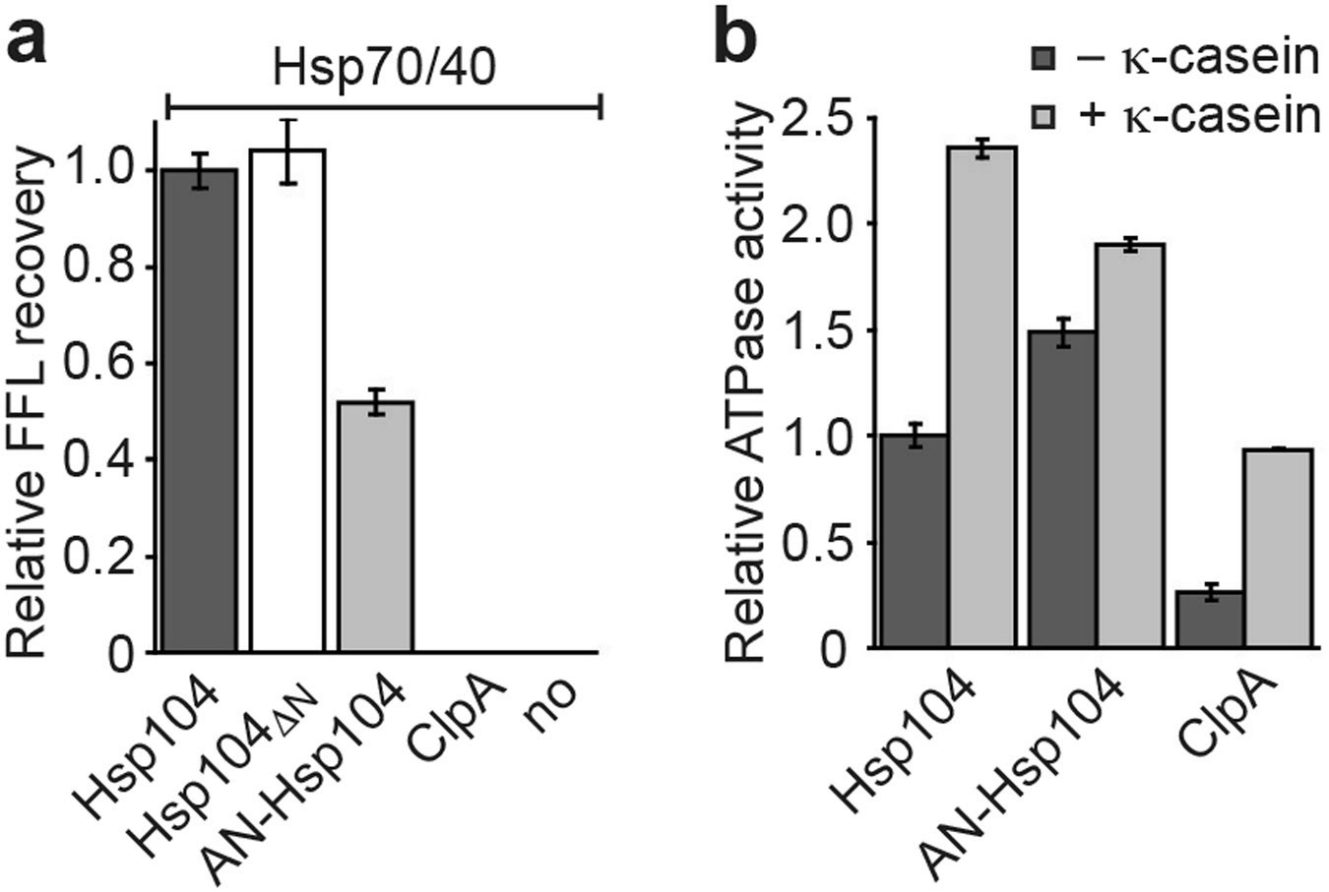
The Hsp104 N domain affects protein disaggregation when present. (**a**) Recovery of FFL activity by Hsp104, Hsp104_ΔN_, AN-Hsp104, and ClpA in the presence of Hsp70/40. (**b**) Basal (black) and κ-casein stimulated (grey) ATPase activities of AN-Hsp104 and ClpA expressed relative to the basal ATPase activity of Hsp104. Averages of three independent measurements ± SD are shown.

Although the Hsp104 N domain is essential for curing yeast prions by Hsp104 overexpression *in vivo* ^16,37^, an Hsp104 variant lacking the N domain (Hsp104_ΔN_) is functional and cooperates with Hsp70/40 in protein disaggregation *in vitro* (Fig. 4a). Because the N domain adopts a stably folded conformation that does not make strong contacts with the D1 domain in the Hsp104 hexamer^11–13^, we asked whether the N domain can be replaced with the structurally homologous N domain of ClpA. ClpA lacks an M domain and does not cooperate with Hsp70/40 in protein disaggregation (Fig. 4a). Instead, ClpA associates with the ClpP protease to degrade ssrA-tagged proteins^46^. However, because the ClpAP system is converted into a protein disaggregase in the presence of the ClpA adaptor ClpS^47^, ClpA must feature, at least in principle, all of the structural elements required for protein disaggregation. We find that the AN-Hsp104 chimera featuring the N domain of *E*. *coli* ClpA reduces protein disaggregation nearly 50% compared to the native Hsp104 bi-chaperone system (Fig. 4a). The latter is not the result of an impaired ATPase activity, which is elevated compared to both Hsp104 and ClpA, and can be further stimulated by κ-casein (Fig. 4b). Hence, the N domain must feature sequence elements that are important to Hsp104’s protein disaggregase function.

### N domain mutants impaired in Hsp70 binding are defective in protein disaggregation

**Figure 5:**
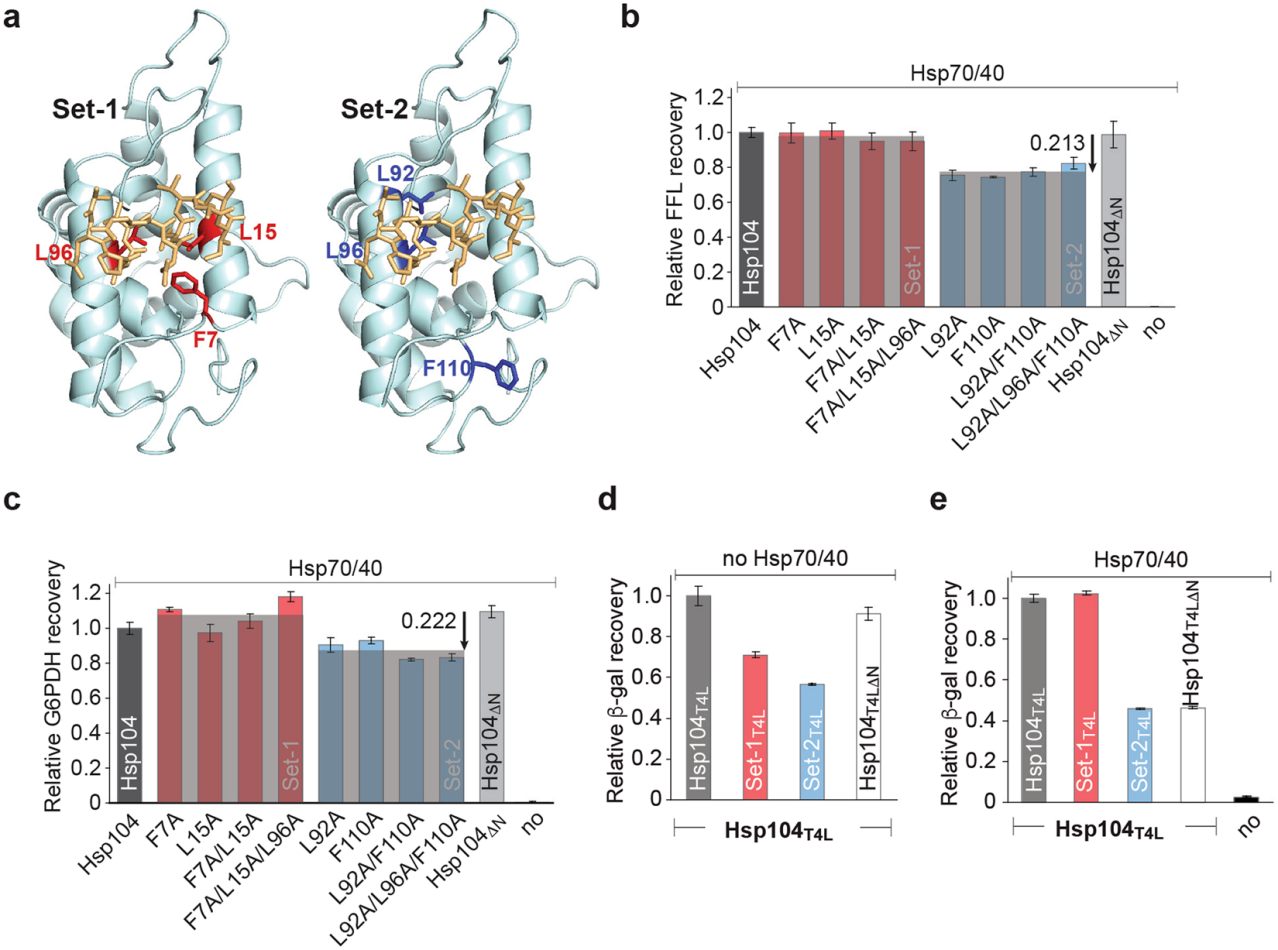
The Hsp104 N domain is bipartite and mediates both substrate binding and Hsp70 interaction. (**a**) Key residues involved in substrate binding are shown in red (Set-1), and those involved in Hsp70 interaction^21^ in blue (Set-2). (**b**,**c**) Set-1 and Set-2 mutants affect protein disaggregation differently. Disaggregase activities of Hsp104 and Hsp104 variants (Set-1 red; and Set-2 blue) in the presence of Hsp70/40. Recoveries of (**b**) FFL and (**c**) G6PDH activity expressed relative to the native bi-chaperone system. (**d**,**e**) Set-1_T4L_ and Set-2_T4L_ show substantial defects in protein disaggregation in a constitutively active background. (**d**) Impaired recovery of β-gal activity by Set-1_T4L_ (red) and Set-2_T4L_ (blue). (**e**) Hsp70/40 restores the protein disaggregation defect of Set-1_T4L_ (red), but not of Set-2_T4L_ (blue). Averages of three independent measurements ± SD are shown.

The crystal structure of Hsp104_ND1_ showed that the N domain contacts the C1 helix of a neighboring molecule (Fig. 2a,b). The observed N domain interactions involve helix A1 (residues 9–24) in the first repeat, and helix A5 (residues 90–105) in the second repeat that overlaps with an Hsp104 segment (residues 91–111) mediating a species-specific interaction with Hsp70^21^. A functional role for helix A1 has not been established for Hsp104, and could facilitate substrate binding. To test this, we selected conserved hydrophobic residues from each interface, Phe7, Leu15, and Leu96 (Set-1), and Leu92, Leu96, and Phe110 (Set-2), mutated them to alanine either alone or in combination, and determined their effect on Hsp104 ATPase activity (see Supplementary Fig. S3). Both sets included Leu96 that is part of a hydrophobic network and likely contributes binding energy (Fig. 5a). Since the N domain does not form strong contacts with the D1 domain, we expect the impact of these mutations to be small. We find that the ATPase activity is unchanged for Set-1 mutants and elevated for Set-2 mutants compared to Hsp104, but below the ATPase activity observed for Hsp104_ΔN_ that lacks the N domain altogether (see Supplementary Fig. S3).

Although it is intriguing that N domain mutations should have any effect on the ATPase activity, comparing the ATPase activity of Hsp104 variants is not a good measure of chaperone function^40^. We therefore evaluated our Set-1 and Set-2 mutants using a coupled-chaperone assay (Fig. 5b,c). To avoid substrate-specific effects, we used chemically denatured firefly luciferase (FFL) and heat-aggregated Glucose-6-phosphate dehydrogenase (G6PDH) as model substrates^11^. We expect that all of our Hsp104 N domain mutants remain functional protein disaggregases because they maintain the critical M domain:Hsp70 interaction required for Hsp104 activation. Although none of the single Set-1 mutants (Hsp104_F7A_ or Hsp104_L15A_) or their double (Hsp104_F7A/L15A_) and triple combination (Set-1) are defective in recovering FFL activity in the presence of Hsp70/40, Hsp104 variants carrying one or more Set-2 mutation (Hsp104_L92A_, Hsp104_F110A_, Hsp104_L92A/F110A_, and Set-2) are substantially impaired (Fig. 5b). The recovery of aggregated FFL by Set-2 mutants is on average ~21.3% less than that observed with Hsp104_ΔN_ suggesting that the N domain contributes to Hsp104’s protein disaggregating activity when present. Similarly, Set-2 but not Set-1 mutants were also impaired in protein disaggregation when using heat-aggregated G6PDH as substrate (Fig. 5c), arguing against a substrate-specific effect. Because residues 91–111 mediate an interaction with the cognate Hsp70 chaperone, the observed protein disaggregation defect of Set-2 mutants is likely the direct result of an impaired interaction between the N domain and Hsp70 needed to facilitate substrate binding or recruitment.

### Set-1 mutants are defective in substrate interaction

The negligible effect on protein disaggregation observed with Set-1 mutants in the presence of Hsp70/40 was unexpected (Fig. 5b,c). Because Hsp70 binding is essential to unleash the protein disaggregating activity of Hsp104, Hsp70/40 may have masked any N domain defects in substrate binding in the coupled-chaperone assay. To test this, we introduced the Set-1 (F7A/L15A/L96A) and Set-2 (L92A/L96A/F110A) triple mutations into the constitutively active Hsp104_T4L_ variant that recovers functional protein in the absence of Hsp70/40^7,11^. Uncoupling the Hsp70/40 requirement showed a clear defect in protein disaggregation (Fig. 5d). Both Set-1_T4L_ and Set-2_T4L_ are substantially impaired in protein disaggregation compared to Hsp104_T4L_, and have a 28.9% (Set-1_T4L_) and 43.5% reduced activity (Set-2_T4L_), respectively, in recovering heat-aggregated β-gal activity (Fig. 5d). In the presence of Hsp70/40, the ability of Set-1_T4L_ to recover functional β-gal is restored to Hsp104_T4L_ levels (Fig. 5e), whereas β-gal recovery by Set-2_T4L_ remains similar to the level observed with Hsp104_T4LΔN_ lacking the N domain (Fig. 5e). These findings suggest that Set-1 mutants are defective in substrate binding, a defect that is rescued in the presence of the Hsp70 system.

### The N domain modulates Hsp104 function

A functional role of the N domain in substrate binding has been reported for ClpB^27,29,36,48^. However, because of an inability to uncouple the Hsp70 requirement from protein disaggregation in the Hsp104:Hsp70/40 bi-chaperone system, these studies could not differentiate between defects in substrate binding and defects in Hsp70 interaction, not to mention defective Hsp70-mediated substrate binding.

**Figure 6:**
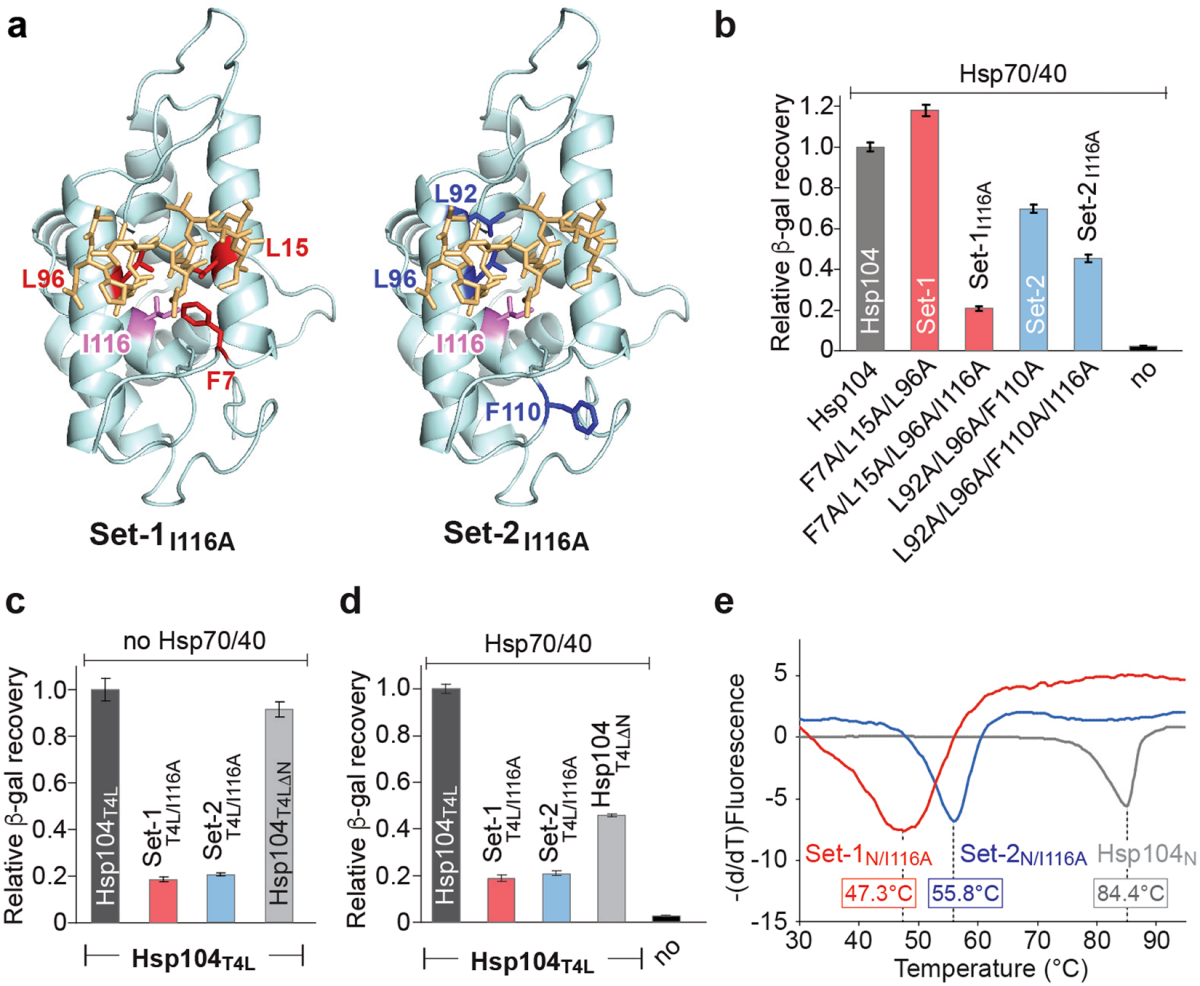
Addition of the I116A mutation to Set-1 and Set-2 triple mutants abrogates protein disaggregation. (**a**) Ribbon diagram of the Set-1_I116A_ and Set-2_I116A_ mutants with Ile116 shown in pink. (**b**) Recovery of β-gal activity by Set-1 and Set-1_I116A_ (red bars), and Set-2 and Set-2_I116A_ (blue bars). (**c**,**d**) Neither Set-1_T4L/I116A_ (red bars) nor Set-2_T4L/I116A_ (blue bars) promotes protein disaggregation without (**c**) or with Hsp70/40 (**d**). Averages of three independent measurements ± SD are shown in Fig. 6b–d. (**e**) The Set-1_N/I116A_ and Set-2_N/I116A_ quadruple mutants have reduced protein stability. Thermal shift assay with the isolated N domain (Hsp104_N_, grey curve) and its Set-1 (Set-1_N/I116A_, red curve) and Set2 variants (Set-2_N/I116A_, blue curve). Representative curves from three independent measurements together with the calculated *T*_m_ values are shown.

It was recently reported that a *T*. *thermophiles* ClpB variant featuring four alanine substitutions in the N domain abrogates protein disaggregation by the bacterial bi-chaperone system^36^. These four residues correspond to Phe7, Leu15, Leu96, and Ile116 in yeast Hsp104 and are identical to the Set-1 triple mutant with the addition of the Ile116 to alanine mutation. Neither Hsp104_I116A_ (see Supplementary Fig. S4) nor the Set-1 triple mutant shows impairment in protein disaggregation (Fig. 5b,c). We therefore introduced the I116A mutation into the Set-1 and Set-2 variants resulting in Set-1_I116A_ and Set-2_I116A_, respectively (Fig. 6a). Interestingly, the quadruple Set-1_I116A_ mutant nearly abolished protein disaggregation (Fig. 6b) underscoring the structural and functional conservation between the bacterial and yeast bi-chaperone system. Similarly, the quadruple Set-2_I116A_ mutant further reduced the activity of the Set-2 triple mutant (Fig. 6b). Because none of the Set-1 single, double, or triple mutants are defective, the combined effect of all four mutations is necessary to abrogate protein disaggregation of Set-1_I116A_ (Figs 5b,c and 6b and Supplementary Fig. S4).

A defect was also observed with Set-1_T4L/I116A_ that is uncoupled from the Hsp70/40 requirement (Fig. 6c). Unlike the Set-1_T4L_ mutant, addition of Hsp70/40 chaperones could not restore the protein disaggregase activity of the Set-1_T4L/I116A_ mutant (Fig. 6d). Addition of the I116A mutation to Set-2_T4L_ further reduced the chaperone activity of the Set-2_T4L_ variant both in the absence and presence of Hsp70/40 (compare Fig. 5d,e with Fig. 6c,d). These findings indicate that the addition of the I116A mutation to Set-1 or Set-2 affected both mutants similarly.

The Hsp104_ND1_ crystal structure showed that Ile116 is part of a hydrophobic core that stabilizes the N domain (Fig. 6a). Therefore, mutating Ile116 to alanine could affect N domain stability. To test this, we bacterially expressed and purified the isolated N domain (Hsp104_N_) and its Set-1_N_ and Set-2_N_ mutants, alone and in combination with I116A, then determined their protein stability by dye binding using differential scanning fluorimetry. The melting temperatures (*T*_m_) of the quadruple Set-1_N/I116A_ and Set-2_N/I116A_ mutants are severely left shifted compared to Hsp104_N_, decreasing the apparent *T*_m_ by 37.1 °C and 28.6 °C, respectively (Fig. 6e), with the addition of the I116A mutation contributing as much as 19.4 °C (see Supplementary Fig. S5). Thus, addition of the I116A mutation to the Set-1 or Set-2 triple mutants destabilizes the N domain and likely inactivated the Hsp104 motor irreversibly possibly by blocking the axial channel.

## Discussion

Hsp100 protein disaggregases are powerful molecular machines that harness the energy derived from ATP binding and hydrolysis to disaggregate a wide variety of aggregated proteins. The crystal structure of Hsp104_ND1_ supports a role for the N domain in binding of a substrate mimic (Fig. 2b). We observe only hydrophobic interactions that would be compatible with accommodating different protein substrates of diverse size, shape, and sequence. Furthermore, the N domain is bipartite and mediates the direct interaction with both substrate and Hsp70, albeit through distinct binding interfaces (Fig. 5a). Although mutations in either interface interfere with protein disaggregation, the underlying mechanism is different. While helix A1 mutants are defective in substrate binding (Fig. 5d), mutations in helix A5 are also impaired in Hsp70 interaction (Fig. 5e). Notably, helix A1 mutants are indistinguishable from Hsp104 in protein disaggregation in the presence of the Hsp70 system (Fig. 5b,c and e), suggesting that Hsp70/40 chaperones can compensate for N domain mutants defective in substrate binding.

Our observations support the notion that some Hsp104 substrates are recognized and bound directly by the Hsp104 motor, while others may depend on Hsp70/40 to be recruited. Thus, in addition to its role as an Hsp104 activator, Hsp70 may serve as an adaptor to increase the Hsp104 substrate spectrum. Our findings also resolve the outstanding question regarding the functional role of the N domain. Although the N domain is dispensable for protein disaggregation by the bi-chaperone system, the N domain facilitates substrate binding and modulates Hsp104 function when present^7,16^. Thus, we propose that lack of an N domain or Hsp104 N domain mutants defective in substrate binding are masked by the presence of the Hsp70 system needed for Hsp104 activation and function. Simultaneously, Hsp104-specific substrates that require a direct, physical interaction with the N domain, such as the prion form of yeast Sup35^15^, may depend on the N domain and cannot be rescued by Hsp70/40 chaperones^16,37^. Together, these findings provide a molecular explanation for the perplexing N domain requirements in distinct Hsp104 activities.

## Methods

### Cloning

*E*. *coli* ClpA was cloned into pET24b. *S*. *cerevisiae* Hsp104_MMM_ and Hsp104 mutants (F7A, L15A, L92A, L96A, F110A, I116A, and their combinations) were generated by QuikChange site-directed mutagenesis. Corresponding Hsp104_T4L_ mutants were generated by cassette mutagenesis. To generate AN-Hsp104, the Hsp104 N domain (M1-L164) was replaced with the equivalent N domain of *E*. *coli* ClpA (M1-E166) and cloned into pProEX HTb. Isolated N domain constructs consisting of the first 165 residues (Hsp104_N_) were PCR amplified and cloned into pProEX HTb.

### Protein expression and purification

Protein constructs were overexpressed in *E*. *coli* BL21-CodonPlus (DE3)-RIL cells (Agilent Technologies, Santa Clara, CA). Protein expression was induced with 0.5 or 1 mM IPTG for 4 h hours at 37 °C (Hsp104_ND1_), for 2 h at 37 °C (ClpA), for 4 h at 30 °C (His_6_-Hsp104_N_ and His_6_-Ydj1), 6 h at 25 °C (His_6_-hHsp70, His_6_-Hsp104_ΔN_, His_6_-Hsp104, and His_6_-Hsp104 mutants), or for 16 h at 16 °C (His_6_-AN-Hsp104, His_6_-Hsp104_T4LΔN_, His_6_-Hsp104_T4L_ and His_6_-Hsp104_T4L_ mutants). SeMet-labeled Hsp104_MMM_ was prepared by transforming an *E*. *coli* methionine auxotroph strain, B834 (DE3) pLysS. Cells were grown in defined medium supplemented with 50 mg/L seleno-DL-methionine. Preparation of SeMet-labeled Hsp104_MMM_ was otherwise identical to Hsp104_ND1_^49^. Cells were lysed using a microfluidizer in buffer A (25 mM Tris-HCl pH 7.5, 300 mM NaCl, 5% glycerol and 5 mM β-mercaptoethanol) containing 30 mM imidazole followed by ultracentrifugation at 45,000 rpm for 1 h at 4 °C. Proteins were purified from cleared lysates by Ni-NTA agarose affinity chromatography. Proteins were eluted in buffer A containing 300 mM imidazole. The N-terminal His_6_-tag was cleaved by incubating the protein with His_6_-TEV protease at 4 °C overnight. The cleaved protein was diluted 1:10 with buffer A and reapplied to a Ni-NTA agarose column to remove the liberated His_6_-tag and His_6_-tagged TEV. Protein concentration was measured by a colorimetric assay using the Protein Assay Dye Reagent (Bio-Rad, Hercules, CA). Hsp104_ND1_/Hsp104_MMM_ was further purified in negative binding mode to a DEAE sepharose column followed by cation exchange chromatography. Hsp104_N_ and its variants were further purified by anion exchange chromatography. AN-Hsp104 and Hsp104_T4L_ variants were purified similarly to Hsp104 in buffer A containing 10% glycerol using Ni-NTA agarose followed by size-exclusion chromatography on a Superdex 200 10/300 GL column (GE Healthcare Bio-Sciences, Pittsburgh, PA). His_6_-Ydj1 and His_6_-Hsp70 were purified by Ni-NTA affinity chromatography as described^21^. His_6_-Ydj1 was further purified on a TOYOPEARL Butyl-650S column (Tosoh Bioscience LLC, King of Prussia, PA) followed by anion exchange chromatography. His_6_-hHsp70 was further purified by anion exchange chromatography.

### Protein crystallization and data collection

Native crystals were grown at 4 °C by mixing 2 µl of protein solution (19 mg/ml) with 1 µl of reservoir solution containing 33% PEG 400 (v/v), 50 mM HEPES-HCl pH 7.2, and 150 mM sodium formate^49^. Crystals were harvested into reservoir solution and flash frozen in liquid nitrogen without additional cryo-protectant. Crystals belonged to space group *P*6_5_22 with unit cell dimensions of *a* = *b* = 179.1 Å, *c* = 69.7 Å, and one molecule per asymmetric unit. Crystals of SeMet-labeled Hsp104_MMM_ protein were grown at 4 °C by the hanging-drop vapor diffusion method by mixing 2 µl of protein (20 mg/ml) in 50 mM Tris-HCl, pH 7.6, 100 mM NaCl, 10 mM MgCl_2_ and in the presence or absence of 5 mM nucleotide with 2 µl of reservoir solution consisting of 19% PEG4000 (w/v), 100 mM MES, pH 6.5, and 20 mM sodium formate. Crystals were harvested into reservoir solution containing 25% (v/v) glycerol and flash frozen in liquid nitrogen. A three wavelength MAD experiment was performed by collecting data near the absorption peak and the edge of selenium, 0.9789 and 0.9792 Å, corresponding to the maximum *f*″ and the minimum *f*′, respectively, and at a low energy remote of 1.0024 Å. Data sets were obtained from a single crystal and were processed using the HKL2000 software package^50^ (Table 1). A total of six selenium sites were identified from the anomalous difference map that was sufficient to solve the structure of Hsp104_MMM_ to 3.5 Å resolution. The resulting phases were used to calculate the electron density map to build the atomic model. Multiple rounds of refinement were carried out using CNSsolve v.1.1^51^ combined with manual rebuilding using COOT^52^ (Table 1, see Supplementary Fig. S2). Atomic coordinates and structure factors for Hsp104_ND1_ have been deposited with the RCSB under accession code 6AMN.

### Biolayer interferometry

Biolayer interferometry was used to examine N domain interaction with peptide, and was performed using an Octet RED96 instrument (Pall FortéBio LLC, Fremont, CA). Biotinylated C1 peptide (Biotin-Ahx-GEVAEPSVRQTVAILRGLQ) was synthesized commercially (AAPPTec LLC, Louisville, KY) and immobilized onto streptavidin-coated biosensors at a concentration of 0.5 µM in binding buffer (50 mM Tris-HCl pH 8.5, 20 mM ammonium citrate, 10% PEG4000 (w/v), and 0.1 mg/ml BSA). Biosensors were immersed in a 200 µl protein solution for 10 min to allow for association and subsequently transferred into 200 µl binding buffer to follow dissociation for 10 min. Binding curves were obtained at 25 °C at different Hsp104_N_ concentrations or BSA control in a 96-well black plate under rocking. Curve fitting and analysis were performed using the GraphPad Prism software (GraphPad Software Inc., La Jolla, CA).

### Chaperone activity assay

To prepare aggregates of model substrates, firefly luciferase (FFL; 10 µM) was denatured in 7 M urea in refolding buffer (25 mM HEPES-KOH pH 7.5, 150 mM potassium acetate, 10 mM magnesium acetate, and 10 mM DTT) for 30 min at 22 °C. β-galactosidase (β-gal; 0.4 µM) was thermally aggregated in refolding buffer for 40 min at 59 °C. Glucose-6-phosphate dehydrogenase (G6PDH; 200 µM) was thermally aggregated in denaturation buffer (4 M urea, 8% glycerol, and 20 mM DTT) for 5 min at 47 °C, diluted 10-fold in refolding buffer, and treated for 1 min at 47 °C.

Chemically denatured FFL was diluted 125-fold in refolding buffer containing the bi-chaperone system (1 μM Hsp104, 1 μM hHsp70, 1 μM Ydj1), 5 mM ATP, and an ATP regenerating system consisting of 25 mM phosphoenolpyruvate and 2 μM pyruvate kinase. Heat-aggregated G6PDH (2.5 µM) and β-gal (0.2 µM) were mixed with the bi-chaperone system (1 µM each) together with 4 mM ATP, 20 mM phosphoenolpyruvate, and 2 μM pyruvate kinase. The activities of recovered model substrates were measured as described^21,40^. G6PDH activity was measured at 30 °C in 50 mM Tris-HCl pH 8.0, 5 mM MgCl_2_, 0.5 mM NAD, and 2 mM glucose-6-phosphate by monitoring the formation of NADH at 340 nm using a UV-1601 spectrophotometer (Shimadzu Corporation, Kyoto, Japan).

### ATPase activity assay

Hsp104 and variants (0.5 µM monomer) or Hsp104_T4L_ and variants (0.2 µM monomer) were incubated with 2 mM ATP at 22 °C for 15 min in the absence or presence of 0.2 mg/ml κ-casein. The amount of released inorganic phosphate was measured using the malachite green assay^53^.

### Thermal shift assay

Thermal shift assays were performed in 396-well thin-wall PCR plates. Purified Hsp104_N_, Set-1_N_, Set-2_N_, Set-1_N/I116A_, and Set-2_N/I116A_ were diluted in buffer consisting of 25 mM HEPES-KOH pH 7.5, 150 mM potassium acetate, and 10 mM DTT. To measure protein stability, 4 mg/ml protein was used per well together with 1x SYPRO Orange (Invitrogen Corporation, Carlsbad, CA). PCR plates were sealed with optical seal, shaken, and centrifuged at 4000 rpm for 2 min after adding the protein. Thermal scanning (20 °C to 95 °C at 0.06 °C/sec) was performed using a Roche Lightcycler 480 (Roche Diagnostics Corporation, Indianapolis, IN). Fluorescence intensity was measured every 1 sec at an excitation wavelength of 465 nm and an emission wavelength of 580 nm. Curve fitting and melting temperature calculations were performed according to manufacturer’s instruction.

## Electronic supplementary material

Supplementary Figures
