# Supplementary material for: Overlapping and Specific Functions of the Hsp104 N Domain Define Its Role in Protein Disaggregation
Source: Sci Rep. 2017 Sep 11;7:11184. doi: 10.1038/s41598-017-11474-9 (PMC5593927; doi:10.1038/s41598-017-11474-9)
Supplement: Supplementary file 1 — Supplementary Figures [file 41598_2017_11474_MOESM1_ESM.pdf]

SUPPLEMENTARY INFORMATION

Overlapping and Specific Functions of the Hsp104 N Domain Define Its Role in Protein Disaggregation

Jungsoon Lee[1], Nuri Sung[1], Jonathan M. Mercado[2], Corey F. Hryc[3], Changsoo Chang[6], Sukyeong Lee[1,5*], and Francis T.F. Tsai[1,2,3,4,5*]

[1]Verna and Marrs McLean Department of Biochemistry and Molecular Biology, [2]Department of Molecular and Cellular Biology, [3]Graduate Program in Structural and Computational Biology and Molecular Biophysics, [4]Department of Molecular Virology and Microbiology, [5]Center for Drug Discovery, Baylor College of Medicine, One Baylor Plaza, Houston, Texas 77030; [6]Structural Biology Center, Biosciences Division, Argonne National Laboratory, Argonne, IL 60439, USA

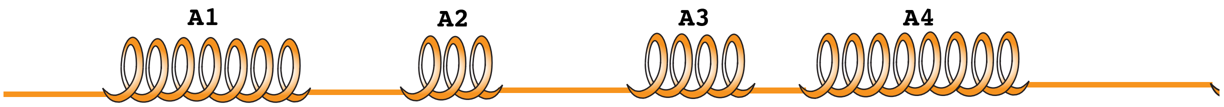


```
Sc_104   1 MNDQTQFTERALTILTLAQKLASDHQHPQLQPIHILAAFIETP-EDGSVPYLQNLIEKGRYDYDLFK----KVVNRNLVRIPQQQPAPAEITPSY 90
Sp_104   1 -MADYPFTDKAAKTLSDAYSIAQSYGHSQLTPIHIAAALLSDSDSNG-TTLLRTIVDKAGGDGQKFE----RSVTSRLVRLPAQDPPPEQVTLSP 89
Ca_104   1 ---MEDFTDNAIKIINNATELAKQQANSQLLPLHFLAAFIPSDDTEGSTQYLKTLVKRARYEWGDFE----RIVNRHLVKIPSQNPPPDEIRPSY 88
Tt_clb   1 -MNLERWTQAAREALAQAQVLAQRMKHQAIDLPHLWAVLLKDERS-----LAWRLLEKAGADPKALK----ELQERELARLPKVEGAEVGQYLTS 85
Ec_clb   1 -MRLDRLTNKFQLALADAQSLALGHDNQFIEPLHLMSALLNQEGG-----SVSPLLTSAGINAGQLR----TDINQALNRLPQVEGTGGDVQPSQ 85
Ec_cla   1 -----MLNQELELSLNMAFARAREHRHEFMTVEHLLLALLSNPSAREA-------LEACSVDLVALRQELEAFIEQTTPVLPASEE-ERDTQPTL 82
```

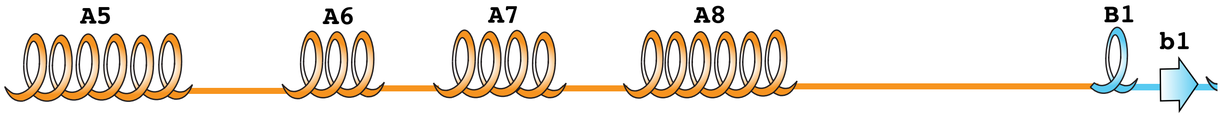


```
Sc_104  91 ALGKVLQDAAKIQKQQKDSFIAQDHILFALFN-DSS-IQQIFKEAQVD-IEAIKQQALELRGNTR-------IDSRGADTNTPLEYLSKYAIDMT 175
Sp_104  90 ESAKLLRNAHELQKTQKDSYIAQDHFIAVFTK-DDT-LKSLLAEAGVT-PKAFEFAVNNVRGNKR-------IDSKNAE--EGFDALNKFTVDLT 172
Ca_104  89 QAGQVLTKANKIKQQQKDSYVAQDHILLALLE-DQS-IKDIFKEAGMS-VDTIKTQAIELRGSQR-------IDSRQADSSSSYEFLNKYCEDFT 173
Tt_clb  86 RLSGALNRAEALMEELKDRYVAVDTLVLALAEATPG-LPG---------LEALKGALKELRGGRT-------VQTEHAE--STYNALEQYGIDLT 161
Ec_clb  86 DLVRVLNLCDKLAQKRGDNFISSELFVLAALESRGT-LADILKAAGAT-TANITQAIEQMRGGES-------VNDQGAE--DQRQALKKYTIDLT 169
Ec_cla  83 SFQRVLQRAVFHVQSSGRNEVTGANVLVAIFSEQESQAAYLLRKHEVSRLDVVNFISHGTRKDEPTQSSDPGSQPNSEEQAGGEERMENFTTNLN 177
```

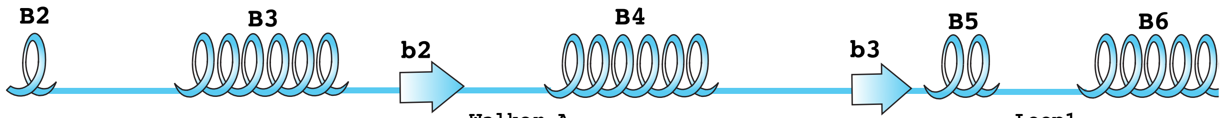


```
                                                 Walker A                                  Loop1
Sc_104 176 EQARQGKLDPVIGREEEIRSTIRVLARRIKSNPCLIGEPGIGKTAIIEGVAQRIIDDDVPTILQGAKLFSLDLAALTAGAKYKGDFEERFKGVLK 270
Sp_104 172 ELARNGQLDPVIGREDEIRRTIRVLSRRTKNNPVLIGEPGVGKTSIAEGLARRIIDDDVPANLSNCKLLSLDVGSLVAGSKFRGEFEERIKSVLK 267
Ca_104 174 EKAREGKIDPVIGREEEIRRVIRVLARRSKSNSVLIGDAGVGKTSIVEGVAQRIVDGDVPNVLAGSRLFALDLGALTAGAKYKGEFEERLKGVLN 268
Tt_clb 162 RLAAEGKLDPVIGRDEEIRRVIQILLRRTKNNPVLIGEPGVGKTAIVEGLAQRIVKGDVPEGLKGKRIVSLQMGSLLAGAKYRGEFEERLKAVIQ 256
Ec_clb 170 ERAEQGKLDPVIGRDEEIRRTIQVLQRRTKNNPVLIGEPGVGKTAIVEGLAQRIINGEVPEGLKGRRVLALDMGALVAGAKYRGEFEERLKGVLN 264
Ec_cla 178 QLARVGGIDPLIGREKELERAIQVLCRRRKNNPLLVGESGVGKTAIAEGLAWRIVQGDVPEVMADCTIYSLDIGSLLAGTKYRGDFEKRFKALLK 272
```

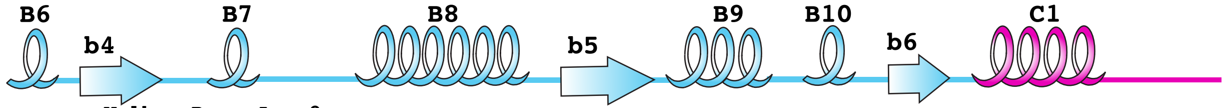


```
               Walker B      Loop2                                 1                        R
Sc_104 271 EIEESKTLIVLFIDEIHMLMGNGK-----DDAANILKPALSRGQLKVIGATTNNEYRSIVEKDGAFERRFQKIEVAEPSVRQTVAILRGLQPKYE 360
Sp_104 268 EVEESETPIILFVDEMHLLMGAGSGGEGGMDAANLLKPMLARGKLHCIGATTLAEYKKYIEKDAAFERRFQIILVKEPSIEDTISILRGLKEKYE 362
Ca_104 269 EIEKSKEFIILFIDEIHMLMGDGK-----SDAANLLKPMLARGALHCIGATTFAEYRKFISKDGAFERRFQKIDVPAATVQETVAILRGIQPKYE 358
Tt_clb 257 EVVQSQGEVILFIDELHTVVGAGK-AEGAVDAGNMLKPALARGELRLIGATTLDEYRE-IEKDPALERRFQPVYVDEPTVEETISILRGLKEKYE 349
Ec_clb 265 DLAKQEGNVILFIDELHTMVGAGK-ADGAMDAGNMLKPALARGELHCVGATTLDEYRQYIEKDAALERRFQKVFVAEPSVEDTIAILRGLKERYE 358
Ec_cla 273 QLEQ-DTNSILFIDEIHTIIGAGAASGGQVDAANLIKPLLSSGKIRVIGSTTYQEFSNIFEKDRALARRFQKIDITEPSIEETVQIINGLKPKYE 366
```

Supplementary Figure S1. Multiple sequence alignment for the N domain and extended D1-large domain of *S. cerevisiae* Hsp104, *S. pombe* Hsp104, *C. albicans* Hsp104, *T. thermophilus* ClpB, *E. coli* ClpB, and *E. coli* ClpA. Residues conserved across species are highlighted in green. Secondary structure elements are labeled in upper case (α-helices) or lower case letters (β-strands), and are colored according to their domain assignment: N domain (A) orange, D1-large domain (B) blue, and D1-small domain (C) magenta. Residues belonging to the Walker A and Walker B motifs are shaded in grey. The sensor-1 (1) and the Arg-finger (R) residues are boxed in blue. A black dot marks every tenth residue.

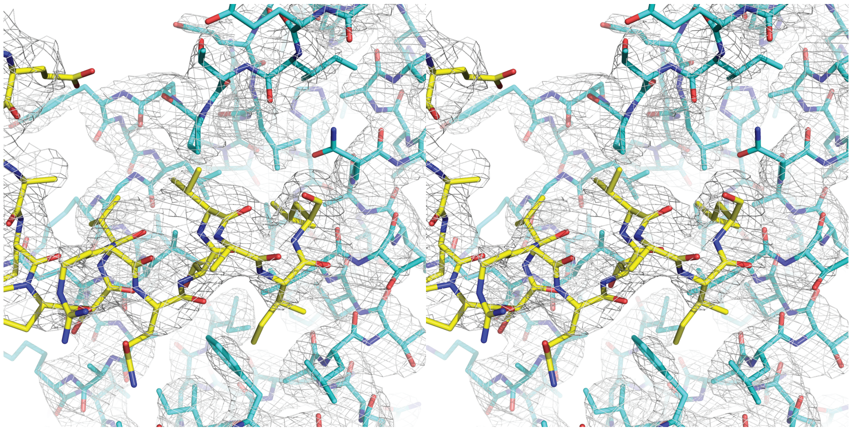

Supplementary Figure S2. Stereo-view of a section of the simulated-annealed composite omit map contoured at the 0.8 σ level. The figure shows the C1 helix (yellow stick model) interacting with the N domain of a neighboring molecule (cyan stick model).

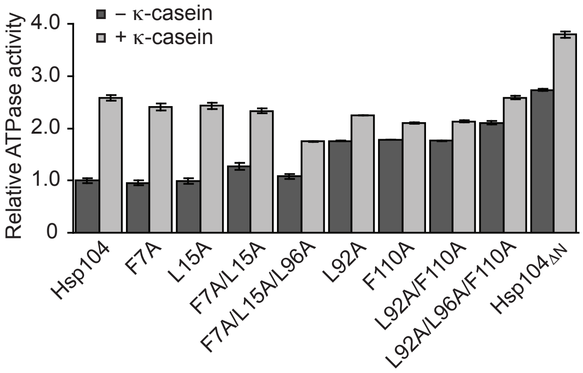

Supplementary Figure S3. The ATPase activity is unaffected for Set-1 mutants and elevated for Set-2 mutants. Basal (black) and κ-casein stimulated (grey) ATPase activities of Hsp104 mutants expressed relative to the basal ATPase activity of Hsp104. Averages of three independent measurements ± SD are shown.

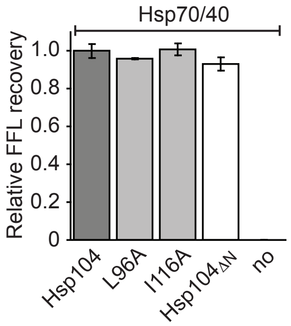

Supplementary Figure S4. The $Hsp104_{L96A}$ and $Hsp104_{I116A}$ single mutants are fully functional in protein disaggregation. Recovery of FFL activity by Hsp104 and Hsp104 variants in the presence of Hsp70/40. Averages of three independent measurements ± SD are shown.

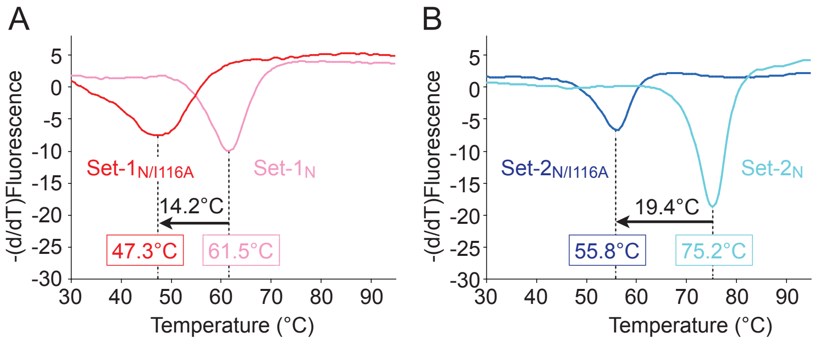

Supplementary Figure S5. Thermal shift assay with Set-1_N and Set-2_N variants. Addition of the I116A mutation to the triple mutants further destabilizes the N domain structure. Representative curves from three independent measurements together with the calculated T_m values are shown.
